# Oleic Acid and Octanoic Acid Sensing Capacity in Rainbow Trout *Oncorhynchus mykiss* Is Direct in Hypothalamus and Brockmann Bodies

**DOI:** 10.1371/journal.pone.0059507

**Published:** 2013-03-22

**Authors:** Marta Librán-Pérez, Marcos A. López-Patiño, Jesús M. Míguez, José L. Soengas

**Affiliations:** Laboratorio de Fisioloxía Animal, Departamento de Bioloxía Funcional e Ciencias da Saúde, Facultade de Bioloxía, Universidade de Vigo, Vigo, Spain; St. Joseph's Hospital and Medical Center, United States of America

## Abstract

In a previous study, we provided evidence for the presence in hypothalamus and Brockmann bodies (BB) of rainbow trout *Oncorhynchus mykiss* of sensing systems responding to changes in levels of oleic acid (long-chain fatty acid, LCFA) or octanoic acid (medium-chain fatty acid, MCFA). Since those effects could be attributed to an indirect effect, in the present study, we evaluated *in vitro* if hypothalamus and BB respond to changes in FA in a way similar to that observed *in vivo*. In a first set of experiments, we evaluated in hypothalamus and BB exposed to increased oleic acic or octanoic acid concentrations changes in parameters related to FA metabolism, FA transport, nuclear receptors and transcription factors, reactive oxygen species (ROS) effectors, components of the K_ATP_ channel, and (in hypothalamus) neuropeptides related to food intake. In a second set of experiments, we evaluated in hypothalamus the response of those parameters to oleic acid or octanoic acid in the presence of inhibitors of fatty acid sensing components. The responses observed *in vitro* in hypothalamus are comparable to those previously observed *in vivo* and specific inhibitors counteracted in many cases the effects of FA. These results support the capacity of rainbow trout hypothalamus to directly sense changes in MCFA or LCFA levels. In BB increased concentrations of oleic acid or octanoic acid induced changes that in general were comparable to those observed in hypothalamus supporting direct FA sensing in this tissue. However, those changes were not coincident with those observed *in vivo* allowing us to suggest that the FA sensing capacity of BB previously characterized *in vivo* is influenced by other neuroendocrine systems.

## Introduction

In mammals specialized neurons within the hypothalamus are able to detect changes in plasma levels of long-chain fatty acid (LCFA), but not short-chain (SCFA) or medium-chain (MCFA) FA, thus contributing to nervous control of energy homeostasis [1]. This capacity has been suggested to be achieved through 4 different mechanisms [1], [2], [3], [4] such as i) FA metabolism through inhibition of carnitine palmitoyltransferase 1 (CPT-1) to import FA-CoA into the mitochondria for oxidation; ii) transport through fatty acid translocase (FAT/CD36); iii) FA-induced activation of novel protein kinase C (PKC) isoforms; and iv) mitochondrial production of reactive oxygen species (ROS) by electron leakage resulting in an inhibition of ATP-dependent inward rectifier potassium channel (K_ATP_) activity. Changes in the activity of those systems in mammalian hypothalamus in response to enhanced LCFA levels have been associated, through not completely understood mechanisms [3], [4], with the reduction in food intake through inhibition of the orexigenic factors agouti-related protein (AgRP) and neuropeptide Y (NPY), and the enhancement of the anorexigenic factors pro-opio melanocortin (POMC) and cocaine and amphetamine-related transcript (CART). In addition to feeding, central glucose and FA detection has been related, through vagal and sympathetic outflow, to the regulation of glucose homeostasis by affecting insulin release in pancreas and endogenous glucose production in liver [2] though FA also directly regulate insulin release from pancreatic β-cells [5].

Fish energy metabolism is rather different than that of mammals since most fish are relatively intolerant to glucose, and they rely more on amino acid and lipid metabolism [6], [7], [8]. Furthermore, a reduced food intake has been observed in several fish species fed with lipid-enriched diets or containing high fat stores [9], [10], [11], [12], [13] suggesting that lipid sensor mechanisms regulating food intake may be present in fish. In a previous study in rainbow trout *Oncorhynchus mykiss* [14] we observed that intraperitoneal acute administration of oleic acid (LCFA) or octanoic acid (MCFA) elicited an inhibition in food intake and induced in hypothalamus a response compatible with fatty acid sensing in which fatty acid metabolism, binding to FAT/CD36 and mitochondrial activity were apparently involved, which is similar to that suggested in mammals except for the apparent capacity of rainbow trout to detect changes in MCFA levels. Changes in those hypothalamic pathways can be related to the control of food intake, since food intake was inhibited when FA metabolism was perturbed (using a fatty acid synthetase (FAS) inhibitor) and changes in mRNA levels of specific neuropeptides such as NPY and POMC were also noticed [14]. The results obtained in Brockmann bodies (BB, main accumulation of pancreatic endocrine cells in this species) also suggested the presence of components of putative fatty acid sensing systems based on fatty acid metabolism and binding to FAT/CD36 [14], which could be related to insulin release since hyperinsulinemia has been demonstrated in fish after increasing circulating FA levels [15]. However, we cannot discard that i) those effects of FA on hypothalamus and BB could be attributed to an indirect effect mediated by changes in other endocrine systems, ii) changes were dependent on fatty acid concentration, and iii) the response of BB may be a consequence of vagal and sympathetic outflow from the hypothalamus in a way similar to that described in mammals [16] or to a direct action similar to that observed for the stimulation of insulin release by FA [5]. Furthermore, additional information is required regarding the precise mechanisms through which those systems are informing of changes in FA levels.

Therefore, we aimed to evaluate *in vitro* (in the absence of external influences) whether or not hypothalamus and BB respond to changes in FA concentration in a way similar to that previously observed *in vivo*. Accordingly, in a first set of experiments, we evaluated in hypothalamus and BB exposed to increased oleic acid or octanoic acid concentrations changes in parameters related to: i) FA metabolism, ii) FA transport, iii) nuclear receptors and transcription factors involved in lipid metabolism, iv) a ROS effector, v) components of the K_ATP_ channel, and vi) only in hypothalamus, neuropeptides related to the metabolic control of food intake. In a second set of experiments, we evaluated in hypothalamus *in vitro* the response of those parameters to oleic acid or octanoic acid in the presence of selected inhibitors related to fatty acid sensing components.

## Materials and Methods

### Fish

Rainbow trout (*Oncorhynchus mykiss* Walbaum) were obtained from a local fish farm (A Estrada, Spain). Fish were maintained for 1 month in 100 litre tanks under laboratory conditions and 12L:12D photoperiod in dechlorinated tap water at 15°C. Fish weight was 103±4 g. Fish were fed once daily (09.00 h) to satiety with commercial dry fish pellets (Dibaq-Diproteg SA, Spain; proximate food analysis was 48% crude protein, 14% carbohydrates, 25% crude fat, and 11.5% ash; 20.2 MJ/kg of feed).

### Ethics Statement

The experiments described were carried out in strict accordance with the Guidelines of the European Union Council (2010/63/UE), and of the Spanish Government (RD 1201/2005) for the use of animals in research, and were approved by the Ethics Committee of the Universidade de Vigo.

### Experimental Design

#### Experiment 1: *In vitro* incubation of hypothalamus and BB at increased concentrations of oleic acid or octanoic acid

Freshly obtained tissues were incubated as previously described [17]. Fish were fasted for 24 h before treatment to ensure basal hormone levels were achieved. Every morning of an experiment, fish were dipnetted from the tank, anaesthesized with MS-222 (50 mg·l^–1^) buffered to pH 7.4 with sodium bicarbonate, euthanized by decapitation, and weighed. The hypothalamus and BB were removed and dissected as described previously [17]. Tissues were rinsed with modified Hanks, medium (128 mM NaCl; 3.63 mM KCl, 2.81 mM NaHCO_3_, 0.85 mM CaCl_2_, 0.55 mM MgSO_4_, 0.4 mM KH_2_PO_4_, 0.23 mM Na_2_HPO_4_, 7.5 mM HEPES, 50 U·ml^−1^ penicillin, and 50 µg·ml^−1^ streptomycin sulphate, pH 7.4; referred to a basal medium), sliced on chilled Petri dishes, and placed in a chilled Petri dish containing 100 ml of modified Hanks, medium.g^−1^ tissue that was gassed with 0.5% CO_2_/99.5% O_2_. To ensure adequate mass, tissues were combined from different fish resulting in pools of 3–4 hypothalami and 3–4 BB. Tissues were incubated in 48-well culture plates at 15°C for 1 h with 250 µl of modified Hanks, medium per well containing 25 mg of tissue that was gassed with a 0.5% CO_2_/99.5% O_2_ mixture. Control wells contained modified Hanks, medium with 2 mM D-glucose. Treated wells contained medium at the same glucose concentration and increased concentrations (1, 10 or 100 µM) of oleic acid or octanoic acid. After 1 h incubation, tissues were quickly removed, rinsed, frozen in liquid nitrogen, and stored at −80°C until assayed. FA concentrations were selected based on FA levels assessed in rainbow trout *in vivo* in hypothalamus and BB [14] and plasma [18], [19], [20], as well as by the concentrations used in comparable *in vitro* studies in rainbow trout [21].

On each experiment, one set of 7 tissue pools per tissue was assessed for enzyme activities, another set of 7 tissue pools was used for the assay of tissue metabolites, and another set of 7 tissue pools was used for the assay of mRNA levels. The number of independent experiments (one set of 7 tissue pools each) carried out was five (N = 5).

#### Experiment 2: *In vitro* incubation of hypothalamus with oleic acid or octanoic acid alone or in the presence of inhibitors of different pathways of fatty acid signalling and metabolism

Freshly obtained hypothalamus were obtained and incubated as described in experiment 1. Control wells contained medium with 2 mM D-glucose. Treated wells contained medium at the same glucose concentration and 100 µM oleic acid or 100 µM octanoic acid alone or in the presence of selected inhibitors of parameters related to fatty acid sensing capacity in mammals. These included: 40 µg.ml^−1^ 4-methylene-2-octyl-5-oxotetrahydrofuran-3-carboxylic acid (C75, FAS (fatty acid synthase) inhibitor), 10 µM R(+)-2-[6-(4-chlorophenoxy)hexyl]-oxirane-2-carboxylic acid (etomoxir, CPT1 inhibitor), 1 µM 6-hydroxy-2,5,7,8-tetramethylchroman-2-carboxylic acid (trolox, ROS scavenger), 20 µM methyl (1*R*,2*R*,6*S*)-2-hydroxy-9-(hydroxymethyl)-3-oxabicyclo[4.3.0]nona-4,8-diene-5-carboxylate (genipin, UCP2 (mitochondrial uncoupling protein 2) inhibitor), 500 µM 7-chloro-3-methyl-4*H*-1,2,4-benzothiadiazine 1,1-dioxide (diazoxide, sulfonylurea receptor (SUR-1) antagonist), 5 µM C, *N*-(((2*E*,4*E*,7*E*)-undeca-2,4,7-trienylidene)amino)nitrous amide (triacsin C, acyl-CoA synthetase (ACS) inhibitor), 50 nM sulfo-N-succinimidyl oleate (SSO, FAT/CD36 inhibitor), and 40 µg.ml^−^15-(tetradecyloxy)-2-furoic acid (TOFA, acetyl CoA carboxylase (ACC) inhibitor). Reagents were previously dissolved in modified Hanks, medium (etomoxir), ethanol (trolox), or DMSO (c75, TOFA, diazoxide, triacsin C, SSO, and genipin); no effects were observed due to the vehicle alone (data not shown). The concentrations of the different agents used were those previously used in fish [17], [21], [22] and mammals [1], [23], [24]. After 1 h incubation, tissues were quickly removed, rinsed, frozen in liquid nitrogen, and stored at −80°C until assayed.

On each experiment, one set of 19 tissue pools per tissue (1 control, 1 oleic acid alone, 1 octanoic acid alone, 8 oleic acid plus different agents, 8 octanoic acid plus different agents) was assessed for enzyme activities, another set of 19 tissue pools was used for the assay of tissue metabolites, and another set of 19 tissue pools was used for the assay of mRNA levels. The number of independent experiments (one set of 19 tissue pools each) carried out was five (N = 5).

### Assessment of Metabolite Levels and Enzyme Activities

Samples used to assess metabolite levels were homogenized immediately by ultrasonic disruption in 7.5 vol of ice-cooled 0.6 M perchloric acid, and neutralized (using 1 M potassium bicarbonate). The homogenate was centrifuged (10,000 g), and the supernatant used to assay tissue metabolites. FA, total lipids, and triglyceride levels were determined enzymatically using commercial kits (Wako Chemicals for FA, and Spinreact for total lipids and triglyceride) adapted to a microplate format.

Samples for enzyme activities were homogenized by ultrasonic disruption with 9 vols ice-cold-buffer consisting of 50 mM Tris (pH 7.6), 5 mM EDTA, 2 mM 1,4-dithiothreitol, and a protease inhibitor cocktail (Sigma). The homogenate was centrifuged (10,000 g) and the supernatant used immediately for enzyme assays. Enzyme activities were determined using a microplate reader INFINITE 200 Pro (Tecan) and microplates. Reaction rates of enzymes were determined by the increase or decrease in absorbance of NAD(P)H at 340 nm or 5,5′-Dithiobis(2-nitrobenzoic acid)-CoA complex (DTNB CoA complex) at 412 nm. The reactions were started by the addition of supernatant (15 µl) at a pre-established protein concentration, omitting the substrate in control wells (final volume 265–295 µl), and allowing the reactions to proceed at 20°C for pre-established times (3–10 min). Enzyme activities are expressed in terms of mg protein. Protein was assayed in triplicate in homogenates using microplates according to the bicinchoninic acid method with bovine serum albumin (Sigma) as standard. Enzyme activities were assessed at maximum rates by preliminary tests to determine optimal substrate concentrations. ATP-citrate lyase (ACLY, *EC* 4.1.3.8), FAS (*EC* 2.3.1.85), and hydroxyacil-CoA dehydrogenase (HOAD, *EC* 1.1.1.35) activities were determined as described previously [25], [26], [27]. CPT (*EC* 2.3.1.21) activity was assessed in a tris-HCl buffer (75 mM, pH 8.0) containing 1.5 mM EDTA, 0.25 mM DTNB, 0.035 mM palmitoyl CoA and 0.7 (hypothalamus) or 2 (BB) mM L-carnitine (omitted for controls).

### mRNA Abundance Analysis by Real-time Quantitative RT-PCR

Total RNA was extracted from tissues (approx. 20 mg) using Trizol reagent (Life Technologies) and treated with RQ1-DNAse (Promega). Two µg total RNA were reverse transcribed into cDNA using Superscript II reverse transcriptase (Life Technologies) and random hexaprimers (Life Technologies). Gene expression levels were determined by real-time quantitative RT-PCR (q-PCR) using the iCycler iQ™ (BIO-RAD). Analyses were performed on 1 µl cDNA using the MAXIMA SYBR® Green qPCR Mastermix (Fermentas), in a total PCR reaction volume of 25 µl, containing 50–500 nM of each primer. mRNA abundance of ACC, ACLY, CART, FAT/CD36, CPT1, citrate synthase (CS), FAS, inward rectifier K^+^ channel pore type 6.-like (Kir6.x-like), liver X receptor α (LXRα), malonyl CoA dehydrogenase (MCD), NPY, POMC, peroxisome proliferator-activated receptor type α (PPARα), sterol regulatory element-binding protein type 1c (SREBP1c), sulfonylurea receptor-like (SUR-like), and UCP2a were determined as previously described in the same species [20], [26], [28], [29], [30], [31], [32], [33], [34]. Sequences of the forward and reverse primers used for each gene expression are shown in Table 1. Relative quantification of the target gene transcripts was done using β-actin gene expression as reference, which was stably expressed in this experiment.

**Table 1:** Nucleotide sequences of the PCR primers used to evaluate mRNA abundance by RT-PCR (qPCR).

	Forward primer	Reverse primer	Annealingtemperature (°C)	Accession Number(GenBank or others)
β-actin	GATGGGCCAGAAAGACAGCTA	TCGTCCCAGTTGGTGACGAT	59	NM_ 001124235.1
ACC	TGAGGGCGTTTTCACTATCC	CTCGATCTCCCTCTCCACT	59	tcbk0010c.b.21_5.1.om.4-(Sigenae)
ACLY	CTGAAGCCCAGACAAGGAAG	CAGATTGGAGGCCAAGATGT	60	CA349411.1
CART	ACCATGGAGAGCTCCAG	GCGCACTGCTCTCCAA	60	NM_001124627
CPT1a	TCGATTTTCAAGGGTCTTCG	CACAACGATCAGCAAACTGG	55	AF327058
CPT1b	CCCTAAGCAAAAAGGGTCTTCA	CATGATGTCACTCCCGACAG	55	AF606076
CPT1c	CGCTTCAAGAATGGGGTGAT	CAACCACCTGCTGTTTCTCA	59	AJ619768
CPT1d	CCGTTCCTAACAGAGGTGCT	ACACTCCGTAGCCATCGTCT	59	AJ620356
CS	GGCCAAGTACTGGGAGTTCA	CTCATGGTCACTGTGGATGG	55	TC89195 (Tigr)
FAS	GAGACCTAGTGGAGGCTGTC	TCTTGTTGATGGTGAGCTGT	59	tcab0001c.e.06 5.1.s.om.8 (Sigenae)
FAT/CD36	CAAGTCAGCGACAAACCAGA	ACTTCTGAGCCTCCACAGGA	62	AY606034.1 (DFCI)
Kir6.x-like	TTGGCTCCTCTTCGCCATGT	AAAGCCGATGGTCACCTGGA	60	CA346261.1.s.om.8∶1:773∶1 (Sigenae)
LXRα	TGCAGCAGCCGTATGTGGA	GCGGCGGGAGCTTCTTGTC	62	FJ470291
MCD	TCAGCCAGTACGAAGCTGTG	CTCACATCCTCCTCCGAGTC	60	BX869708.s.om.10 (Sigenae)
NPY	CTCGTCTGGACCTTTATATGC	GTTCATCATATCTGGACTGTG	58	NM_001124266
POMC	CTCGCTGTCAAGACCTCAACTCT	GAGTTGGGTTGGAGATGGACCTC	60	TC86162 (Tigr)
PPARα	CTGGAGCTGGATGACAGTGA	GGCAAGTTTTTGCAGCAGAT	55	AY494835
SREBP1c	GACAAGGTGGTCCAGTTGCT	CACACGTTAGTCCGCATCAC	60	CA048941.1
Sur-like	CGAGGACTGGCCCCAGCA	GACTTTCCACTTCCTGTGCGTCC	62	tcce0019d.e.20_3.1.s.om.8 (Sigenae)
UCP2a	TCCGGCTACAGATCCAGG	CTCTCCACAGACCACGCA	57	DQ295324

ACC, Acetyl-CoA carboxylase; ACLY, ATP-citrate lyase; CART, cocaine- and amphetamine-related transcript; CPT1, carnitine palmitoyl transferase type 1; CS, citrate synthetase; FAS, fatty acid synthetase; FAT/CD36, fatty acid translocase; Kir6.x-like, inward rectifier K^+^ channel pore type 6.-like; LXRα, liver X receptor α; MCD, malonyl CoA dehydrogenase; NPY, neuropeptide Y; POMC, pro-opio melanocortin; PPARα, peroxisome proliferator-activated receptor type α; SREBP1c, sterol regulatory element-binding protein type 1c; SUR-like, sulfonylurea receptor-like; UCP2a, mitochondrial uncoupling protein 2a.

Thermal cycling was initiated with incubation at 95°C for 15 min using hot-start iTaq™ DNA polymerase activation; 40 steps of PCR were performed, each one consisting of heating at 95°C for 15 s for denaturing, annealing at specific temperatures (Table 1) for 30 s, and extension at 72°C for 30 s. Following the final PCR cycle, melting curves were systematically monitored (55°C temperature gradient at 0.5°C/s from 55 to 95°C) to ensure that only one fragment was amplified. Each sample was analyzed in triplicate. All the replicates of each sample were located in the same plate for each gene to allow comparisons. We included in all the plates the standard curve (by triplicate), and controls for NTC and RT negative control (by duplicate). Only efficiency values between 85–100% were accepted (the R^2^ for all the genes assessed was always higher than 0.985). Relative quantification of the target gene transcript with the β-actin reference gene transcript was made following the Pfaffl method [35].

### Statistics

Comparisons among groups were carried out using Student t test (paired comparisons) or one-way ANOVA (multiple comparisons) followed by a Student-Newman-Keuls test, and differences were considered statistically significant at *P*<0.05. When necessary data were log transformed to fulfill the conditions of the analysis of variance.

## Results

### Experiment 1: *In vitro* Incubation of Hypothalamus and BB at Increased Concentrations of Oleic Acid or Octanoic Acid

**Figure 1:**
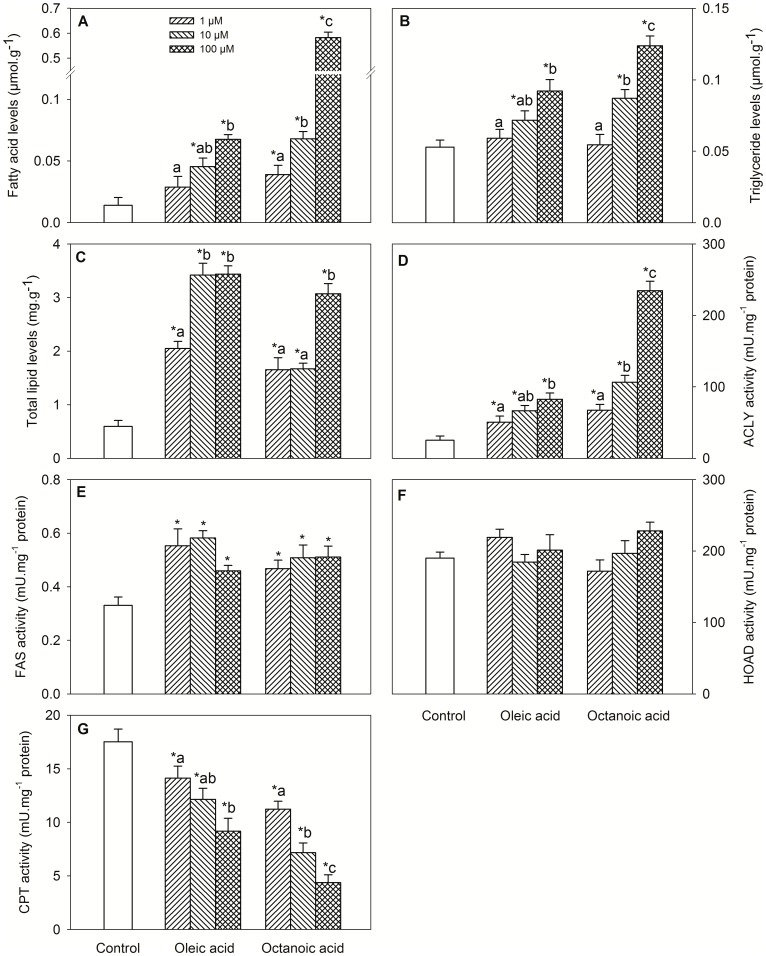
Levels of metabolites and enzyme activities in hypothalamus incubated with oleic acid or octanoic acid. Levels of fatty acid (A), triglyceride (B) and total lipid (C), and activities of ACLY (D), FAS (E), HOAD (F), and CPT (G) in hypothalamus of rainbow trout incubated *in vitro* for 1 h at 15°C in modified Hanks’ medium containing 2 mM D-glucose alone (control) or containing 1, 10, or 100 µM oleic acid or octanoic acid. Each value is the mean+S.E.M. of 5 independent experiments carried out with pools of hypothalami from 3–4 different fish. *, significantly different (P<0.05) from control fish. Different letters indicate significant differences (P<0.05) among fatty acid concentration within each fatty acid treatment.

Changes in metabolite levels and enzyme activities assessed in hypothalamus are shown in Fig. 1. Treatment with oleic acid or octanoic acid elicited increased levels of FA (Fig. 1A, dose-response), triglycerides (Fig. 1B, dose-response) and total lipids (Fig. 1C), and activities of ACLY (Fig. 1D, dose-response) and FAS (Fig. 1E), and decreased CPT activity (Fig. 1G, dose-response). No significant differences were noticed for HOAD activity (Fig. 1F).

Changes in mRNA abundance in hypothalamus are shown in Table 2. A down-regulation after oleic acid or octanoic acid treatment was observed for mRNA levels of FAT/CD36 (dose-response for oleic acid), ACC, ACLY (dose-response), CS, FAS (dose-response), UCP2a, Kir6.x-like (dose-response for octanoic acid), SUR-like (dose-response for oleic acid), LXRα (dose-response), and only after oleic acid treatment for NPY (dose-response). An up-regulation after oleic acid or octanoic acid treatment was observed for CPT1c, MCD (dose-response), PPARα (dose-response), CART (dose-response for oleic acid), and POMC (dose-response for octanoic acid), and only after octanoic acid treatment for CPT1d and SREBP1c.

**Table 2:** mRNA levels in hypothalamus of rainbow trout incubated *in vitro* for 1 h at 15°C in modified Hanks’ medium containing 2 mM D-glucose alone (control) or containing 1, 10, or 100 µM oleic acid or octanoic acid.

	Oleic acid (µM)			Octanoic acid (µM)		
	1	10	100	1	10	100
*Fatty acid transport*						
FAT/CD36	−1.78*a	−2.21*ab	−2.94*b	−2.56*a	−3.12*b	−3.03*ab
*Fatty acid metabolism*						
ACC	−1.13a	−1.85*b	−1.57*b	−1.33*a	−1.37*a	−1.69*b
ACLY	−1.05a	−1.41*ab	−1.79*b	−1.13	−1.48*ab	−2.03*b
CPT1c	−1.03a	+1.06a	+1.48*b	+1.59*	+1.74*	+1.43*
CPT1d	−1.06	−1.02	+1.01	+5.01*	+4.98*	+4.82*
CS	−1.81*	−1.98*	−2.12*	−1.54*	−1.81*	−1.56*
FAS	+1.04a	−1.22ab	−1.43*b	−1.13a	−1.18a	−1.46*b
MCD	+1.02a	+1.71*b	+2.61*b	+1.25a	+1.87*b	+2.03*b
*Mitochondrial uncoupling*						
UCP2a	+1.00a	−2.12*b	−2.22*b	−1.85*	−1.97*	−2.42*
*K_ATP_ channel*						
Kir6.x-like	−1.33*	−1.42*	−1.66*	−1.47*a	−1.82*ab	−2.50*b
SUR-like	−1.16a	−1.62*b	−1.80*b	+1.07a	−1.48*b	−1.39*b
*Transcription factors*						
LXRα	−1.35*a	−1.49*a	−1.87*b	−1.23a	−1.37*a	−1.64*b
PPARα	+1.04a	+1.32*b	+1.41*b	+1.01a	+1.41*b	+1.50*b
SREBP1c	+1.17	+1.01	+1.02	+1.58*	+1.44*	+1.56*
*Food intake control*						
CART	+1.31a	+1.44*ab	+1.78*b	+1.19	+1.39*	+1.57*
NPY	−1.14a	−1.39*a	−1.98*b	−1.08	+1.07	+1.12
POMC	+1.55*	+1.43*	+1.49*	+1.51*a	+1.73*ab	+2.32*b

Each value is the mean of 5 independent experiments carried out with pools of hypothalami from 3–4 different fish. Data is expressed as fold-induction (+, increase; −, decrease) with respect to the control group (expression results were normalized by β-actin mRNA levels, mRNA levels-no variation). *, significantly different (P<0.05) from control fish. Different letters indicate significant differences (P<0.05) among concentration within each fatty acid treatment.

**Figure 2:**
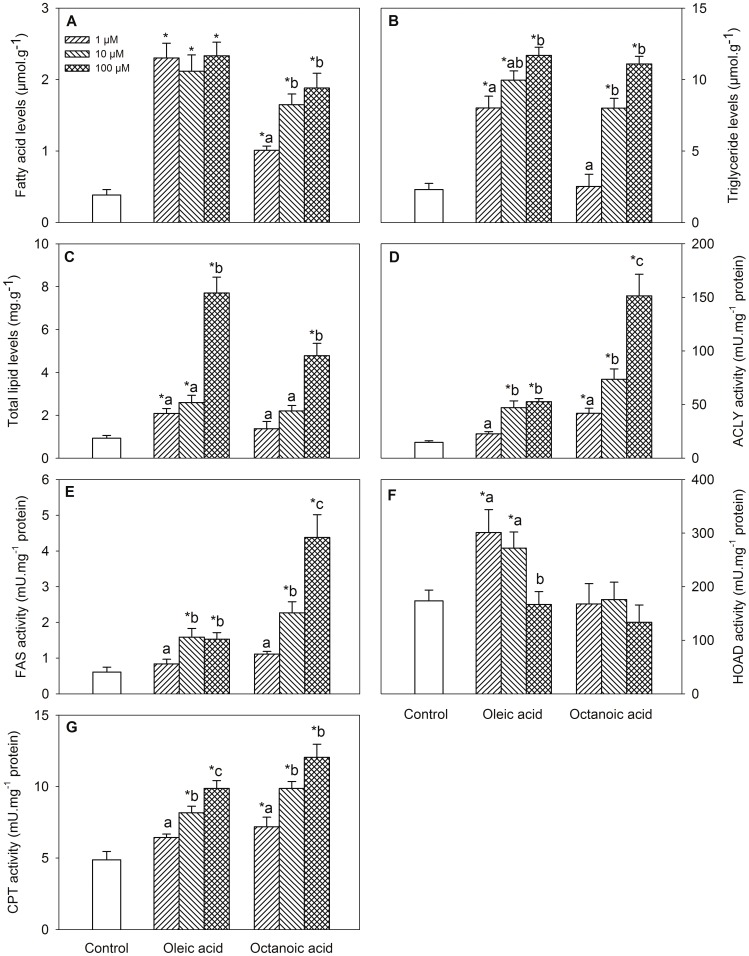
Levels of metabolites and enzyme activities in Brockmann bodies incubated with oleic acid or octanoic acid. Levels of fatty acid (A), triglyceride (B) and total lipid (C), and activities of ACLY (D), FAS (E), HOAD (F), and CPT (G) in Brockmann bodies of rainbow trout incubated *in vitro* for 1 h at 15°C in modified Hanks’ medium containing 2 mM D-glucose alone (control) or containing 1, 10, or 100 µM oleic acid or octanoic acid. Each value is the mean+S.E.M. of 5 independent experiments carried out with pools of Brockmann bodies from 3–4 different fish. *, significantly different (P<0.05) from control fish. Different letters indicate significant differences (P<0.05) among fatty acid concentration within each fatty acid treatment.

Changes in metabolite levels and enzyme activities assessed in BB are shown in Fig. 2. Treatment with oleic acid or octanoic acid elicited increased levels of FA (Fig. 2A, dose-response for octanoic acid), triglycerides (Fig. 2B, dose-response) and total lipids (Fig. 2C, dose-response), and activities of ACLY (Fig. 2D, dose-response), FAS (Fig. 2E, dose-response for octanoic acid), and CPT (Fig. 2G), and only after oleic acid treatment for HOAD activity (Fig. 2F).

Changes in mRNA abundance in BB are shown in Table 3. A down-regulation after oleic acid or octanoic acid treatment was observed for mRNA levels of ACC (dose-response for octanoic acid), ACLY (dose-response for oleic acid), Kir6.x-like (dose-response for oleic acid), SUR-like (dose-response), PPARα and SREBP1c, and only after octanoic acid treatment for CPT1a. An up-regulation after oleic acid or octanoic acid treatment was observed for CPT1b, CS, FAS (dose-response for oleic acid), only after oleic acid treatment for CPT1a, and only after octanoic acid treatment for LXRα (dose-response). mRNA levels of FAT/CD36 after treatment with any of the fatty acids and mRNA levels of MCD after oleic acid treatment displayed changes at only one fatty acid concentration.

**Table 3:** mRNA levels in Brockmann bodies of rainbow trout incubated *in vitro* for 1 h at 15°C in modified Hanks’ medium containing 2 mM D-glucose alone (control) or containing 1, 10, or 100 µM oleic acid or octanoic acid.

	Oleic acid (µM)			Octanoic acid (µM)		
	1	10	100	1	10	100
*Fatty acid transport*						
FAT/CD36	+1.20a	+1.98*b	−1.17a	−1.49*	−1.33	−1.07
*Fatty acid metabolism*						
ACC	−1.47*a	−1.45*a	−2.70*b	−1.65*a	−2.08*ab	−2.57*b
ACLY	−1.24a	−1.38*a	−1.87*b	−1.53*	−1.59*	−1.41*
CPT1a	+1.78*a	+4.68*b	+2.08*a	−3.45*a	−1.60*b	−2.32*b
CPT1b	+1.07a	+1.61*b	+1.79*b	+1.61*	+2.32*	+1.85*
CS	+1.39*	+1.42*	+1.63*	+2.11*	+2.27*	+2.18*
FAS	+1.16a	+2.75*b	+3.29*b	+1.06a	+1.58*b	+1.52*b
MCD	−1.09a	−1.12a	−2.16*b	+1.08	+1.09	+1.26
*K_ATP_ channel*						
Kir6.x-like	−1.04a	−1.39*b	−1.55*b	−1.11a	−2.23*b	−2.03*b
SUR-like	−1.03a	−1.51*b	−1.79*b	−1.16a	−1.56*b	−2.56*c
*Transcription factors*						
LXRα	−1.11	−1.08	+1.10	+1.86*a	+2.06*a	+2.59*b
PPARα	−1.87*ab	−1.59*a	−2.47*b	−1.79*	−1.52*	−1.83*
SREBP1c	−4.18*	−4.99*	−4.70*	−3.97*	−4.43*	−3.53*

Each value is the mean of 5 independent experiments carried out with pools of Brockmann bodies from 3–4 different fish. Data is expressed as fold-induction (+, increase; −, decrease) with respect to the control group (expression results were normalized by β-actin mRNA levels, mRNA levels-no variation). *, significantly different (P<0.05) from control fish. Different letters indicate significant differences (P<0.05) among concentration within each fatty acid treatment.

### Experiment 2: In vitro Incubation of Hypothalamus with Oleic Acid or Octanoic Acid alone or in the Presence of Inhibitors of Different Pathways of Fatty Acid Signalling and Metabolism

In all cases, the effects of oleic acid or octanoic acid alone compared with controls were similar than those assessed in the first experiment. Considering that we evaluated treatment with oleic acid or octanoic acid alone or in the presence of 8 different inhibitors, representing with standard figures all the results obtained in this experiment would mean the use of an unmanageable amount of multiple figures. Therefore, in favour of simplicity, the effects of inhibitors are summarized in Table 4 describing in which cases the presence of the inhibitor significantly (P<0.05) counteracted the effects produced by the treatment with 100 µM oleic acid or 100 µM octanoic acid alone. HOAD activity, for which no significant differences were found in experiment 1 was not assessed in this experiment.

**Table 4:** Response of metabolite levels, enzyme activities, and mRNA abundance of several parameters related to fatty acid sensing in hypothalamus of rainbow trout incubated *in vitro* for 1 h at 15°C in modified Hanks’ medium containing 100 µM oleic acid (Ol) or 100 µM octanoic acid (Oc) alone (controls) or 100 µM oleic acid or 100 µM octanoic acid and selected inhibitors related to fatty acid sensing capacity in mammals.

			Inhibitors													
	C75		Etomoxir		Trolox		Genipin		Diazoxide		Triacsin C		SSO		TOFA	
Parameters	Ol	Oc	Ol	Oc	Ol	Oc	Ol	Oc	Ol	Oc	Ol	Oc	Ol	Oc	Ol	Oc
*Metabolite levels*																
Fatty acid																
Triglyceride		+		+		+		+	+		+		+		+	
Total lipid	+	+		+		+	+	+				+				
*Enzyme activities*																
ACLY	+	+	+	+											+	+
CPT	+	+	+	+							+	+			+	
FAS	+	+	+	+		+		+	+							+
*mRNA abundance*																
FAT/CD36	+								+		+		+	+	+	
ACC	+	+		+											+	+
ACLY									+		+	+				
CPT1c			+	+							+	+			+	
CPT1d												+				
CS		+	+	+							+	+			+	+
FAS	+		+					+			+	+			+	
MCD	+	+	+	+					+		+	+			+	
UCP2a					+	+	+	+	+	+			+			
Kir6.x-like					+		+	+	+	+						+
SUR-like					+	+	+	+	+	+						
LXRα		+	+		+	+		+	+	+			+	+		
PPARα						+	+		+		+		+	+		
SREBP1c					+	+	+	+	+				+	+		
CART												+				
NPY	+			+	+		+		+		+		+		+	+
POMC				+	+	+	+	+	+			+	+		+	+

These included: 40 µg.ml^−1^ C75 (FAS inhibitor), 50 µM etomoxir (CPT1 inhibitor), 1 µM trolox (ROS scavenger), 20 µM genipin (UCP2 inhibitor), 500 µM diazoxide (sulfonyl urea receptor 1 antagonist), 5 µM triacsin C (ACS inhibitor), 50 nM SSO (FAT/CD36 inhibitor), and 40 µg.ml^−1^ TOFA (ACC inhibitor). Only those parameters for which significant effects of oleic acid or octanoic acid treatment alone were noticed (Fig. 1 and Table 2) were evaluated for inhibitor action. Values represent the mean of 5 independent experiments carried out with pools of hypothalami from 3–4 different fish. +, inhibitor significantly (P<0.05) counteracted the effect of oleic acid or octanoic acid alone.

C75, 4-methylene-2-octyl-5-oxotetrahydrofuran-3-carboxylic acid. Etomoxir, R(+)-2-[6-(4-chlorophenoxy)hexyl]-oxirane-2-carboxylic acid). Trolox, 6-hydroxy-2,5,7,8-tetramethylchroman-2-carboxylic acid). Genipin, methyl (1*R*,2*R*,6*S*)-2-hydroxy-9-(hydroxymethyl)-3-oxabicyclo[4.3.0]nona-4,8-diene-5-carboxylate. Diazoxide, 7-chloro-3-methyl-4*H*-1,2,4-benzothiadiazine 1,1-dioxide. Triacsin C, *N*-(((2*E*,4*E*,7*E*)-undeca-2,4,7-trienylidene)amino)nitrous amide). SSO, sulfo-N-succinimidyl oleate. TOFA, 5-(tetradecyloxy)-2-furoic acid.

C75 in the presence of oleic acid or octanoic acid counteracted the effects of FA alone for levels of triglycerides (only octanoic acid) and total lipids, activities of ACLY, CPT, and FAS, and mRNA levels of FAT/CD36 (only oleic acid), ACC, CS (only octanoic acid), FAS (only oleic acid), MCD, LXRα (only octanoic acid), and NPY (only oleic acid).

Etomoxir in the presence of oleic acid or octanoic acid counteracted the effects of FA alone for levels of triglycerides (only octanoic acid) and total lipids (only octanoic acid), activities of ACLY, CPT, and FAS, and mRNA levels of ACC (only octanoic acid), CPT1c, CS, FAS (only oleic acid), MCD, LXRα (only oleic acid), NPY (only octanoic acid), and POMC (only octanoic acid).

Trolox in the presence of oleic acid or octanoic acid counteracted the effects of FA alone for levels of triglycerides and total lipids (only for octanoic acid in both cases), FAS activity (only octanoic acid), and mRNA levels of UCP2a, Kir6.x-like (only oleic acid), SUR-like, LXRα, PPARα (only octanoic acid), SREBP1c, NPY (only oleic acid), and POMC.

Genipin in the presence of oleic acid or octanoic acid counteracted the effects of FA alone for levels of triglycerides (only octanoic acid) and total lipids (only octanoic acid), FAS activity (only octanoic acid), and mRNA levels of FAS (only octanoic acid), UCP2a, Kir6.x-like, SUR-like, LXRα (only octanoic acid), PPARα (only oleic acid), SREBP1c, NPY (only oleic acid), and POMC.

Diazoxide in the presence of oleic acid or octanoic acid counteracted the effects of FA alone for levels of triglycerides (only oleic acid), FAS activity (only oleic acid), and mRNA levels of FAT/CD36, ACLY, MCD (only oleic acid in the three cases), UCP2a, Kir6.x-like, SUR-like, LXRα, PPARα, SREBP1c, NPY, and POMC (only oleic acid in the last four cases).

Triacsin C in the presence of oleic acid or octanoic acid counteracted the effects of FA alone for levels of triglycerides (only oleic acid) and total lipids (only octanoic acid), CPT activity, and mRNA levels of FAT/CD36 (only oleic acid), ACLY, CPT1c, CPT1d (only octanoic acid), CS, FAS, MCD, PPARα (only oleic acid), CART (only octanoic acid), NPY (only oleic acid), and POMC (only octanoic acid).

SSO in the presence of oleic acid or octanoic acid counteracted the effects of FA alone for levels of triglycerides (only oleic acid), and mRNA levels of FAT/CD36, Kir6.x-like (only oleic acid), LXRα, PPARα, SREBP1c, NPY (only oleic acid), and POMC (only oleic acid).

TOFA in the presence of oleic acid or octanoic acid counteracted the effects of FA alone for levels of triglycerides (only oleic acid), activities of ACLY, CPT (only oleic acid), and FAS (only octanoic acid), and mRNA levels of FAT/CD36 (only oleic acid), ACC, CPT1c (only oleic acid), CS, FAS (only oleic acid), MCD (only oleic acid), Kir6.x-like (only octanoic acid), NPY, and POMC.

## Discussion

### The Effects of Oleic Acid or Octanoic Acid on Parameters Related to Lipid Sensing in Hypothalamus in vitro are Similar to those Previously Observed *in vivo*

The treatment with oleic acid or octanoic acid is reflected in the hypothalamus by increased levels of FA, triglycerides and total lipids. These changes support an increased entry of FA that may elicit responses consistent with FA sensing mechanisms. Accordingly, the treatment with both FA elicited in most parameters dose-dependent changes, which were similar for both FA, and also in general similar to those observed previously *in vivo* [14] for levels of FA and total lipids, activities of ACLY, FAS, and HOAD, and mRNA levels of ACLY, FAS, NPY, PPARα, LXRα, CS, Kir6.x-like, and SUR-like, and only after treatment with oleic acid for mRNA levels of CPT1d and POMC. Only few parameters displayed different responses than those previously observed *in vivo* after treatment with both FA, such as for levels of triglycerides and mRNA levels of CART, SREBP1c, and CPT1c. The finding of similar results *in vitro* than those observed *in vivo* allow us to suggest that the action of FA on putative fatty acid sensing mechanisms in hypothalamus of rainbow trout is direct and not mediated by other endocrine systems or nutrients.

In the fatty acid sensing system characterized in mammals based on FA metabolism [3], [4], increased LCFA levels inhibit the ability of hypothalamic CPT1 to import FA-CoA into mitochondria for oxidation, which can lead to inhibition of food intake through effects on the expression of orexigenic and anorexigenic neuropeptides. This system was apparently working in rainbow trout hypothalamus *in vitro* since the most important parameters involved in such mechanism displayed changes compatible with those of the mammalian model, except for the fact that in mammals the system does not respond to MCFA like octanoic acid [4] as it does in rainbow trout. These changes include the decreased mRNA levels of ACLY and CS [36] whereas the decreased mRNA levels of FAS are also in agreement with the pharmacological inhibition observed in mammals [3], [4]. mRNA levels of CPT1c as well as activities of FAS and ACLY did not respond in the same way than in the mammalian model. The changes in FAS and ACLY activities were similar than those previously observed *in vivo* whereas the increase noticed in mRNA levels of CPT1c was unexpected compared with previous *in vivo* results [14]. However, CPT activity (not assessed in our previous *in vivo* study) did show a clear inhibition after treatment with both FA.

The mechanism for FA sensing through FAT/CD36 [1] is also apparently working in hypothalamus of rainbow trout *in vitro* since increased mRNA levels of PPARα and SREBP1c (the later only for octanoic acid) and decreased mRNA levels of LXRα were noted, which are compatible with those observed in the mammalian model [37], [38] though in mammals no effects of octanoic acid are present. The increase observed in mRNA levels of PPARα in parallel with increases in mRNA levels of related genes such as CPT1 and MCD is also in agreement with that observed in liver of rainbow trout [39] and flounder [40]. However, mRNA levels of FAT/CD36 displayed a clear decrease in response to increased FA levels which was different to that previously observed *in vivo* [14].

Another mechanism for lipid sensing in mammals is through mitochondrial production of ROS by electron leakage resulting in an inhibition of K^+^_ATP_ [2]. Hypothalamus of rainbow trout exposed to increased levels of oleic acid or octanoic acid displayed a clear inhibition of mRNA levels of the components of the K^+^_ATP_ channel, which is in agreement with that expected for oleic acid but not octanoic acid in the mammalian model [41]. We also observed a clear down regulation of UCP2a mRNA levels in hypothalamus exposed to oleic acid or octanoic acid in agreement with the known effects of increased ROS levels as described in mammalian models [41]. It is interesting to mention that activated UCP2 may reduce mitochondrial ROS levels in zebrafish brain [42].

The activation of FA sensing mechanisms in mammalian hypothalamus has been related to the control of food intake through not well understood mechanisms involving the control of the expression of anorexigenic (POMC and CART) and orexigenic (AGRP and NPY) factors [3], [4]. In our previous study [14], we observed that the inhibition of food intake in rainbow trout elicited by *in vivo* treatment with oleic (but not octanoic) acid coincided with decreased mRNA levels of NPY and increased levels of POMC. Moreover, Figueiredo-Silva et al. [30] also observed changes in mRNA levels of NPY and CART in hypothalamus of rainbow trout fed a lipid-enriched diet. In the present study, we observed that the presence of oleic acid or octanoic acid stimulated mRNA levels of anorexigenic peptides (POMC and CART) whereas mRNA levels of the orexigenic NPY decreased after oleic acid treatment. The differences with the results obtained *in vivo* could indicate that the enhanced FA levels are inducing changes in the expression *in vitro*, i.e. the mechanisms are present in the tissue and respond to FA, but under *in vivo* conditions other systems are interacting with the expression of those neuropeptides and therefore the response is masked. In any case, changes in mRNA levels of neuropeptides are in agreement with the reduction of food intake addressed *in vivo* for oleic acid [14] lending further support for the physiological role of these lipid sensing systems in rainbow trout. In mammals changes in CPT1c appear to modulate the expression of neuropeptides in hypothalamus [43]. In our previous *in vivo* study there were no effects of octanoic acid treatment on mRNA levels of CPT1c and neuropeptides whereas in the present study both parameters were affected by octanoic acid treatment. Therefore, the FA metabolic sensing system was apparently fully activated by LCFA and MCFA *in vitro* though *in vivo* the responses of neuropeptides were not apparent after MCFA treatment. Therefore, the response *in vivo*, at least for the octanoic acid, displays an interaction with other systems/factors.

### The Effects of Oleic Acid or Octanoic Acid on Parameters Related to Lipid Sensing in Brockmann Bodies *in vitro* are Similar to those Observed in Hypothalamus but Different than those Previously Observed in vivo in Brockmann Bodies

In mammals lipid metabolism in the β-cell is critical for the normal regulation of insulin secretion [44] and FA directly act to regulate insulin release from pancreatic β-cells [5]. In fish, the studies available described that lipid metabolism is indeed important in BB [45], [46] where insulin release is also influenced by circulating FA levels [15], [47].

In the present study, the treatment with oleic acid or octanoic acid elicited increased levels of FA, triglycerides and total lipids in a way similar to that observed in hypothalamus but different than that described in mammals where pancreatic cells do not respond to MCFA such as octanoic acid [44]. Changes observed in BB in metabolite levels, enzyme activities and mRNA levels were in general similar to those observed in hypothalamus thus supporting direct FA sensing in this tissue. However, in contrast to that observed in hypothalamus, changes observed in most parameters assessed *in vitro* in BB were in general different (in certain cases even converse) than those observed previously *in vivo* in the same tissue [14] such as for levels of total lipids, activities of ACLY, FAS (oleic acid), and HOAD (oleic acid), and mRNA levels of ACLY, FAS, SREBP1c, PPARα, LXRα, FAT/CD36 (oleic acid), CS, Kir6.x-like, SUR-like, CPT1a, and CPT1b. Only few parameters displayed similar responses than those previously observed *in vivo* after treatment with oleic acid or octanoic acid [14], such as for levels of FA and triglycerides, and only after octanoic acid treatment for FAS and HOAD activities, and mRNA levels of FAT/CD36. Those responses can be generalized to the three putative FA sensing systems evaluated (FA metabolism, transport through FAT/CD36, and mitochondrial activity).

The finding of such a different response *in vitro* than that observed *in vivo* allow us to suggest that the action of FA on putative fatty acid sensing mechanisms in BB of rainbow trout observed *in vivo* is influenced by other endocrine systems like insulin [15], [47]. However, we may also hypothesize that the *in vivo* response of BB could be a consequence of vagal and sympathetic outflow from the hypothalamus in a way similar to that described in mammals [1].

### The Use of Specific Inhibitors Provide Further Evidence for the Specific Mechanisms of Fatty Acid Sensing in Rainbow Trout Hypothalamus

To obtain additional information regarding the mechanisms through which putative FA-sensing systems might directly inform of changes in FA levels, we incubated hypothalamus *in vitro* with oleic acid or octanoic acid alone or in the presence of specific inhibitors and antagonists of the FA-sensing mechanisms present in mammalian hypothalamus. As for the use of those inhibitors in fish, we previously demonstrated in rainbow trout the effects of C75 and TOFA on food intake [14], and the inhibitory effects of diazoxide in mRNA levels of components of the K^+^_ATP_ channel [17] whereas other studies validated the use of etomoxir [21], trolox [22], SSO [48], and triacsin C [49]. However, there are no references available, as far as we are aware, for the use of genipin in fish. Considering the high number of multiple comparisons carried out it is possible that some of the significant differences observed might be false positives. This fact precluded us for discussing specific actions of all antagonists tested in the response of all parameters assessed. However, several general trends are apparent from the results obtained, and these can be summarized as follows: i) All the antagonists in the presence of oleic acid or octanoic acid counteracted the action of the FA alone for several of the parameters assessed, and for many of the antagonists the action was comparable for oleic acid or octanoic acid with the exception of trolox, which was more effective counteracting the effects of octanoic acid, and diazoxide, which was more effective counteracting the effects of oleic acid. ii) The inhibitors related to FA metabolism such as C75, etomoxir, triacsin C and TOFA generally counteracted the effects of oleic acid or octanoic acid alone in parameters related to that mechanism (levels of metabolites, enzyme activities and mRNA levels of ACC, ACLY, CPT1c, CPT1d, CS, and FAS). Moreover, those inhibitors counteracted few of the effects of oleic acid or octanoic acid alone on parameters related to other FA sensing mechanisms such as those involved in FAT/CD36 transport or mitochondrial activity (mRNA levels of FAT/CD36, UCP2a, Kir6.x-like, SUR-like, LXRα, PPARα or SREBP1c). iii) The inhibitors related to transport through FAT/CD36 and mitochondrial activity such as trolox, genipin, SSO, and diazoxide generally counteracted the effects of oleic acid or octanoic acid alone in related parameters (mRNA levels of FAT/CD36, UCP2a, Kir6.x-like, SUR-like, LXRα, PPARα or SREBP1c). In contrast, few effects of those inhibitors were noted in parameters related to FA metabolism.

There are almost no effects of inhibitors counteracting the effects of oleic acid or octanoic acid in the expression of CART whereas the effects of oleic acid or octanoic acid on expression of NPY and POMC were mainly counteracted by the presence of inhibitors of FA sensing mechanisms acting through FAT/CD36 and mitochondrial activity like trolox, genipin, SSO, and diazoxide. It is interesting to mention that counteractive effects were noted for oleic acid or octanoic acid for POMC whereas for NPY counteractive effects were mainly noted for oleic acid.

These results give therefore further support for the presence of FA sensing mechanisms in rainbow trout hypothalamus similar to those already described in mammals [1], [2], [3], [4] and possibly related to the control of food intake but, in contrast to mammals, with the ability of responding either to MCFA like octanoic acid or LCFA like oleic acid. The similar responses elicited by octanoic acid and oleic acid could be related to the fact that fish are able to store considerable amounts of MCFA [50], [51] and in rainbow trout there is no preferential oxidation of MCFA compared with LCFA [9]. However, since the later study was carried out after dietary replacement of fish oil (rich in LCFA) by coconut oil (rich in MCFA) the possibility of differences existing in the transport and oxidation of LCFA and MCFA cannot be discarded.

### Conclusions

In the present study, we provide information supporting that components of different FA sensing systems are present in rainbow trout hypothalamus displaying dose-dependent changes in response to increased levels of octanoic acid (MCFA) or oleic acid (LCFA). Changes in those parameters are also reflected in the expression of anorexigenic and orexigenic peptides related to the control of food intake. The responses observed *in vitro* in hypothalamus are comparable to those observed in a previous study *in vivo* [14] allowing us to suggest that the increase of circulating LCFA or MCFA levels in rainbow trout is directly sensed in hypothalamus. Further support was obtained by incubating hypothalamus with oleic acid or octanoic acid in the presence of specific inhibitors, whose presence counteracted in many cases the effects of the FA alone. These results give further support to the capacity of rainbow trout hypothalamus to sense changes either in MCFA or LCFA levels through mechanisms related to FA metabolism, binding to FAT/CD36 and mitochondrial activity comparable to those addressed in mammals but with differences in their responses, especially the capacity of responding to octanoic acid in rainbow trout.

The incubation of BB *in vitro* in the presence of increased concentrations of oleic acid or octanoic acid resulted in changes that in general were comparable to those observed in hypothalamus thus supporting the existence of direct FA sensing capacity in this tissue. However, these *in vitro* changes were not coincident with those previously observed *in vivo* in this species [14]. Therefore, we may hypothesize that the FA sensing capacity of BB previously characterized *in vivo* is influenced by other neuroendocrine systems. Therefore, further studies, using intracerebroventricular treatments with oleic acid and octanoic acid are necessary to elucidate if the activation of those sensing systems in BB is a consequence of hypothalamic FA sensing through vagal and sympathetic outflow and/or can be attributed to the interaction with other endocrine systems.
